# Translational Regulation of Clock Genes BMAL1 and REV-ERBα by Polyamines

**DOI:** 10.3390/ijms22031307

**Published:** 2021-01-28

**Authors:** Akihiko Sakamoto, Yusuke Terui, Takeshi Uemura, Kazuei Igarashi, Keiko Kashiwagi

**Affiliations:** 1 Faculty of Pharmacy, Chiba Institute of Science, Choshi, Chiba 288-0025, Japan; asakamoto@cis.ac.jp (A.S.); yterui@cis.ac.jp (Y.T.); 2 Amine Pharma Research Institute, Innovation Plaza at Chiba University, Chiba 260-0856, Japan; uemura@amine-pharma.com (T.U.); iga16077@gmail.com (K.I.); 3 Graduate School of Pharmaceutical Science, Chiba University, Chiba 260-8675, Japan

**Keywords:** polyamine modulon, BMAL1, REV-ERBα, circadian clock, translation

## Abstract

Polyamines stimulate the synthesis of specific proteins at the level of translation, and the genes encoding these proteins are termed as the “polyamine modulon”. The circadian clock generates daily rhythms in mammalian physiology and behavior. We investigated the role of polyamines in the circadian rhythm using control and polyamine-reduced NIH3T3 cells. The intracellular polyamines exhibited a rhythm with a period of about 24 h. In the polyamine-reduced NIH3T3 cells, the circadian period of circadian clock genes was lengthened and the synthesis of BMAL1 and REV-ERBα was significantly reduced at the translation level. Thus, the mechanism of polyamine stimulation of these protein syntheses was analyzed using NIH3T3 cells transiently transfected with genes encoding enhanced green fluorescent protein (EGFP) fusion mRNA with normal or mutated 5′-untranslated region (5′-UTR) of *Bmal1* or *Rev-erbα* mRNA. It was found that polyamines stimulated BMAL1 and REV-ERBα synthesis through the enhancement of ribosomal shunting during the ribosome shunting within the 5′-UTR of mRNAs. Accordingly, the genes encoding *Bmal1* and *Rev-erbα* were identified as the members of “polyamine modulon”, and these two proteins are significantly involved in the circadian rhythm control.

## 1. Introduction

Polyamines (putrescine, spermidine, and spermine) are cationic aliphatic amines present in almost all living organisms [1,2]. Since polyamines mainly exist as a polyamine-RNA complex, the synthesis of specific proteins is stimulated by polyamines [1,3]. We have proposed that a set of genes whose expression is enhanced by polyamines at the level of translation can be classified as a “polyamine modulon”, and thus far have identified 10 different genes in eukaryotes as components of the polyamine modulon [4,5,6,7,8,9,10]. In mammalian cells, to identify the polyamine modulon, polyamine-reduced cells were prepared using α-difluoromethylornithine (DFMO), an inhibitor of ornithine decarboxylase (ODC), and proteins in cells were compared between control and polyamine-reduced cells. Since DFMO is a specific inhibitor of polyamine biosynthesis, polyamines reversed the cell growth [11]. Using DFMO-treated cells, we tried to find “polyamine modulon”, and the mechanism of polyamine stimulation at the level of translation [3]. The mechanisms of polyamine stimulation are as follows. First, polyamines enhance ribosome shunting, which involves discontinuous scanning by 40S ribosomal subunits during the scanning of the 5′ untranslated region (5′-UTR) of mRNA. This was observed in Cct2 (T-complex protein 1, β subunit) and COX4 syntheses [4,5]. Second, in the 5′-UTR of mRNA, a complementary sequence, consisting of more than five nucleotides, to the nucleotide sequences at the 3′-end of 18S rRNA, i.e., a CR sequence (complementary sequence to 18S rRNA), is normally present at −17 to −32 upstream from the initiation codon AUG. When the CR sequence is located more distally from the initiation codon AUG, polyamines cause structural changes in the mRNA and stimulated protein synthesis. This was observed in eEF1A (a translation elongation factor) synthesis [6]. Third, when a microRNA inhibits protein synthesis through the interaction with the complementary sequence in some mRNAs, polyamines release the microRNA from mRNA and stimulate protein synthesis. This was observed in EXT2 (a protein involved in extension of polysaccharide chains) synthesis [7]. Fourth, when an RNA G-quadruplex exists at the 5′-UTR of an mRNA, polyamines stimulate protein synthesis through the unfolding of the G-quadruplex. This was observed in CHSY1 (chondroitin synthase 1) synthesis [8]. Fifth, when a microRNA stimulates protein synthesis, polyamines enhance the interaction between the 5′-UTR of mRNA with the microRNA, resulting in the destabilization of the double-stranded RNA between the 5′-UTR and the open reading frame (ORF) of mRNA. This was observed in GCN5 (histone acetyltransferase in the nuclei) synthesis [10]. Sixth, when the size of 5′-UTR is short, initiation complex formation (AUG-dependent Met-tRNA*i* binding to 80S ribosomes) is stimulated by polyamines. This was observed in HAT1 (histone acetyltransferase in cytoplasm) synthesis [10].

Circadian rhythms affect many physiological functions, including sleep, body temperature, blood pressure, and endocrine and autonomic functions [12,13,14,15]. In mammals, the master pacemaker exists in the hypothalamic suprachiasmatic nucleus (SCN), but the circadian oscillator is also located in peripheral tissues.

The relationship between polyamines and circadian clocks has been reported in several ways. Circadian rhythms of ODC activity and putrescine content are present in rat liver [16]. Expression and activity of the polyamine biosynthetic enzymes ODC, *S*-adenosylmethionine decarboxylase 1 (AdoMetDC1), and spermidine synthase (SRM) are under circadian control, and are non-rhythmic in various clock mutant mice [17,18,19,20]. Consequently, polyamine biosynthesis is regulated by circadian clock genes. On the other hand, polyamines participate in circadian period control in mammalian cells through regulating the interaction of the core clock repressors PER2 and CRY1 [21]. However, the crosstalk between polyamines and circadian clocks has not been clarified in detail.

In this study, we investigated the role of polyamines in the circadian clock at the molecular level using an NIH3T3 cell culture system. The intracellular polyamines exhibited a rhythm with an approximate 24 h period. Polyamine depletion lengthened the circadian period of clock genes. In addition, we found that the synthesis of BMAL1 and REV-ERBα was stimulated by polyamines at the level of translation, and demonstrated that polyamines enhanced ribosome shunting on the 5′-UTR of *Bmal1* and *Rev-erbα* mRNAs. Thus, the genes encoding *Bmal1* and *Rev-erbα* were found to be members of “polyamine modulon”.

## 2. Results

### 2.1. Rhythmicity of Polyamine Contents in NIH3T3 Cells

**Figure 1:**
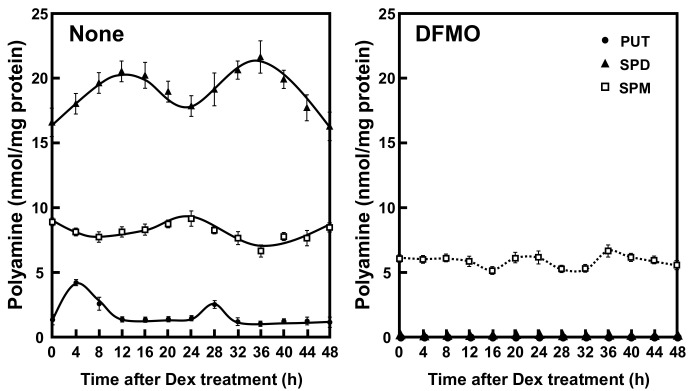
Temporal content profiles of intracellular polyamines in mouse fibroblast NIH3T3 cells. NIH3T3 cells were synchronized by dexamethasone (Dex) and harvested at 4 h intervals throughout a period of 48 h in the presence and absence of α-difluoromethylornithine (DFMO). The levels of intracellular polyamines were measured by HPLC as described in the Materials and Methods section. PUT, putrescine; SPD, spermidine; SPM, spermine.

To investigate the role of polyamines in the circadian clock, we first measured the polyamine contents in NIH3T3 cells whose circadian clock was synchronized with dexamethasone (Dex). This cell line has been routinely used as a model system for circadian rhythmicity. Putrescine (PUT) showed rhythmic peak at 4 h and 28 h, spermidine (SPD) at 12 h and 36 h, and spermine (SPM) at 24 h after Dex treatment (Figure 1). Thus, a rhythm with a 24 h period was observed for the three polyamines—PUT, SPD, and SPM—in cultured cells. In DFMO-treated cells, PUT and SPD were unable to be detected, SPM decreased to about 60%, and the rhythms disappeared. The results support some reports that polyamines are regulated by circadian clock genes and suggest that polyamines regulate the expression of clock genes and functions [16].

### 2.2. Effects of Polyamines on the Expression of Circadian Clock Genes

**Figure 2:**
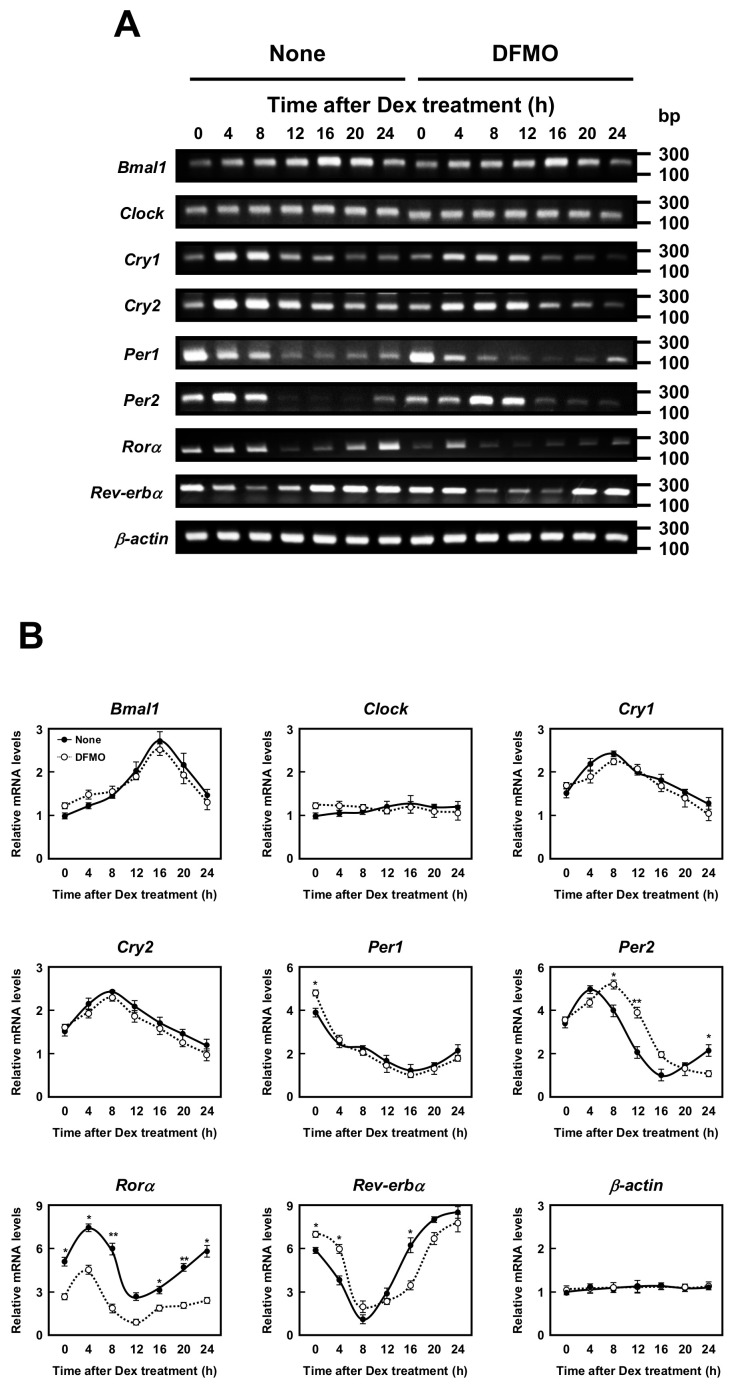
Measurement of mRNA level of circadian clock genes. (**A**,**B**) Temporal mRNA expression profiles of circadian clock genes in control and DFMO-treated NIH3T3 cells were measured by RT-PCR. The levels of circadian genes were normalized to β-actin and presented as relative expression. * *p* < 0.05; ** *p* < 0.01.

The molecular mechanism of the circadian clocks consists of a transcription–translation feedback loop by the clock genes. We next examined whether polyamines affect the expression of circadian clock genes (i.e., *Bmal1*, *Clock*, *Cry1*, *Cry2*, *Per1*, *Per2*, *Rorα*, and *Rev-erbα* genes). As shown in Figure 2, the mRNA levels of eight kinds of circadian clock genes in normal and DFMO-treated cells were measured by semi-qPCR. We have previously shown that the method is quantitative [10]. The levels of these mRNAs except *Rorα* mRNA were nearly equal in both control and DFMO-treated cells. As a control, β-actin was used. However, the level of *Rorα* mRNA in control cells was higher than in DFMO-treated cells. The results suggest that the expression of *Rorα* is regulated by polyamines at the level of transcription. The rhythmic pattern of the circadian clock genes was similar to those reported previously [22,23], however, the period of *Rev-erbα* and *Per2* expression was slightly delayed in DFMO-treated cells.

**Figure 3:**
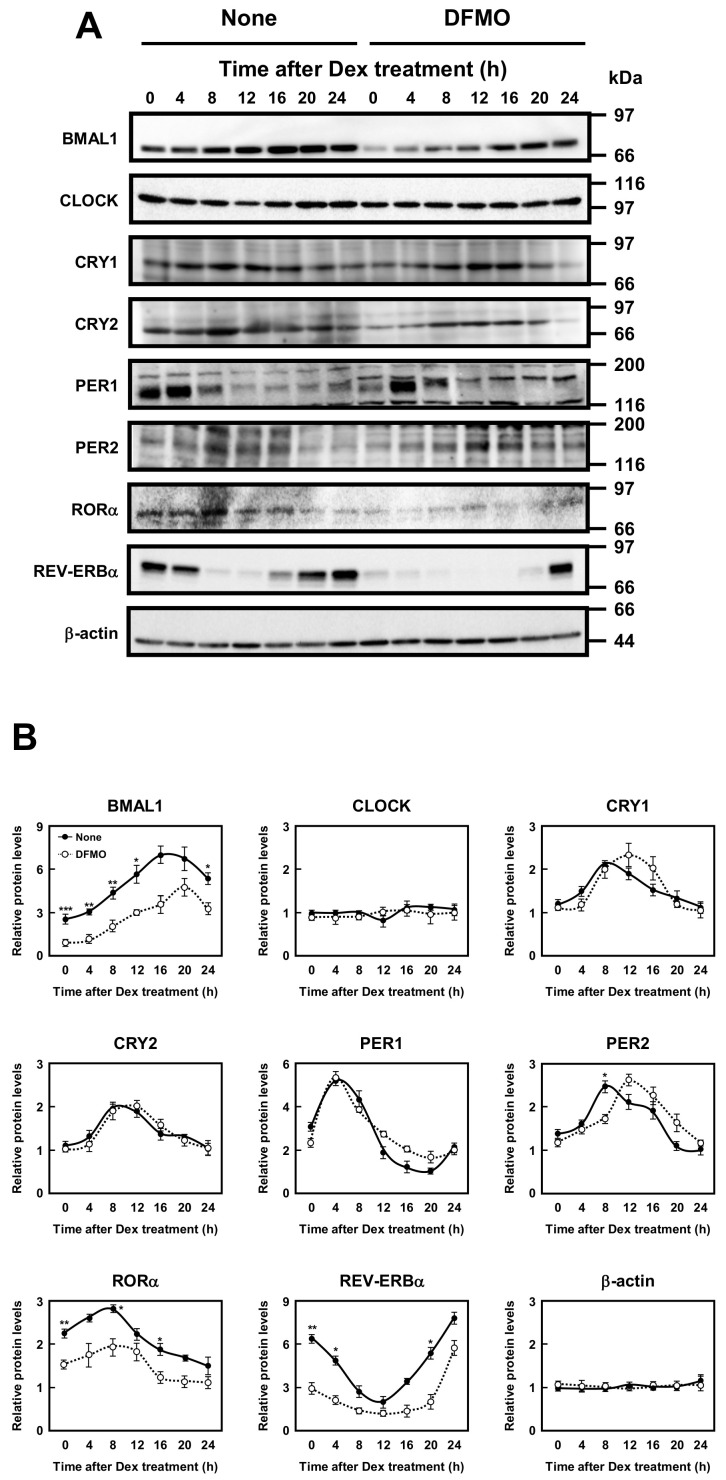
Measurement of protein level of circadian clock genes. (**A**,**B**) Temporal protein expression encoded by the circadian clock genes in control and DFMO-treated NIH3T3 cells were determined by Western blotting. The levels of circadian genes were normalized to β-actin and presented as relative expression. * *p* < 0.05; ** *p* < 0.01; *** *p* < 0.001.

**Figure 4:**
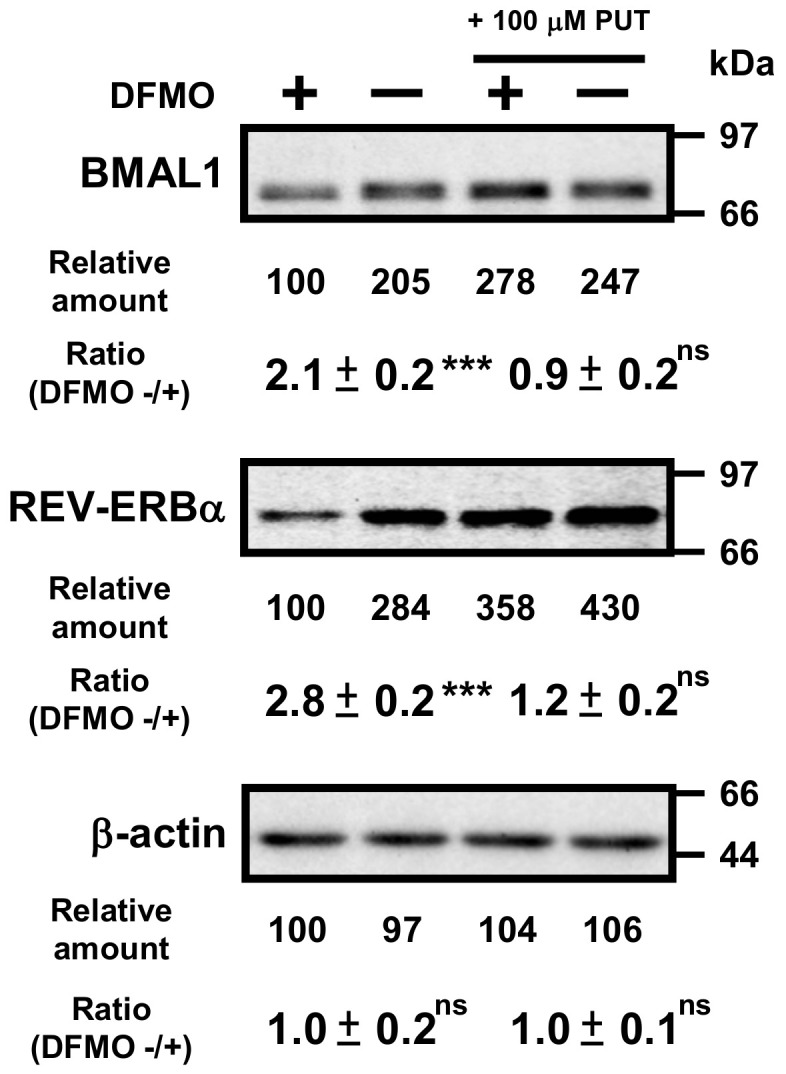
Effects of 1 mM DFMO and/or 100 μM putrescine (PUT) on the expression levels of BMAL1 and REV-ERBα in NIH3T3 cells that were not synchronized by Dex. NIH3T3 cells were cultured in the presence and absence of 1 mM DFMO and/or 100 μM PUT for 24 h. Protein levels of BMAL1 and REV-ERBα were analyzed by Western blotting. As a control, the level of β-actin was examined. ns = not significant; *p* ≥ 0.05; *** *p* < 0.001.

Then, protein levels of circadian clock genes in normal and DFMO-treated cells were measured (Figure 3). Some of the clock protein levels were shifted from mRNA levels by about 4 h. It has been reported that the phase of clock protein rhythms is delayed from the phase of RNA rhythms [24]. The effect of polyamines on the rhythmic patterns of the clock proteins was similar to that of mRNA. For example, the level of CLOCK was unchanged in the presence or absence of DFMO, while the level of RORα was reduced by the decrease in polyamines, similar to the mRNA level. However, the protein levels of BMAL1 and REV-ERBα were significantly decreased in DFMO-treated cells. The degree of polyamine stimulation of BMAL1 and REV-ERBα at each time was about 1.5- to 3-fold (Figure 3B). To confirm that the decrease in BMAL1 and REV-ERBα in DFMO-treated cells is caused by the decrease in polyamines, we measured the levels of BMAL1 and REV-ERBα in DFMO-treated cells that were not synchronized with Dex, and they were also decreased (Figure 4). Furthermore, through the addition of 100 μM PUT to DFMO-treated cells, the levels of BMAL1 and REV-ERBα recovered to normal level (Figure 4). The results suggest that synthesis of BMAL1 and REV-ERBα is enhanced by polyamines at the level of translation, i.e., the genes encoding *Bmal1* (*Arntl*) and *Rev-erbα* (*Nr1d1*) are members of polyamine modulon. In addition, it was suggested that the transcriptional enhancement of *Rorα* by polyamines is caused by translational enhancement of BMAL1 because BMAL1 protein stimulates transcription of *Rorα* by interacting with the E-box of the promoter region [13].

### 2.3. Mechanism of Polyamine Stimulation of BMAL1 and REV-ERBα Synthesis

**Figure 5:**
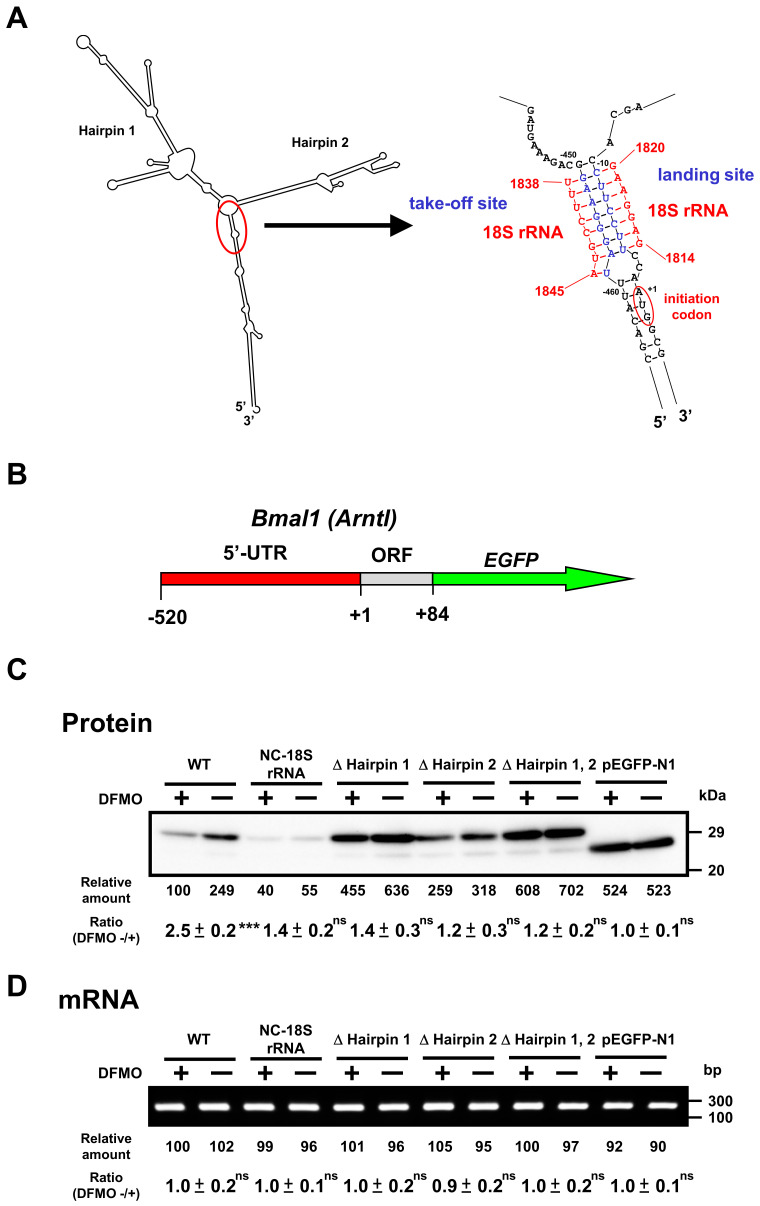
Effect of polyamines on the synthesis of BMAL1-enhanced green fluorescent protein (EGFP) derived from normal and modified *Bmal1-EGFP* mRNAs in NIH3T3 cells. (**A**) Optimal computer folding of the 5′ untranslated region (UTR) of *Bmal1* mRNA was performed by the method of Zuker [26]. Candidates for take-off and landing sites, which are complementary sequence to 18S rRNA are shown in blue, together with the sequence of 18S rRNA in red. (**B**) Structures of *Bmal1-EGFP* fusion genes are shown. (**C**,**D**) The levels of protein and mRNA were measured by Western blotting and RT-PCR, respectively, after transient transfection of *Bmal1-EGFP* fusion gene encoding normal and modified 5′-UTR of *Bmal1* mRNA, 28 amino acids of the amino terminal end of BMAL1 protein, and full length of EGFP protein. As a control, Western blotting and RT-PCR for EGFP were performed. ns *p* ≥ 0.05; *** *p* < 0.001.

To study the mechanism of polyamine stimulation of BMAL1 and REV-ERBα protein synthesis, we employed fusion mRNAs containing a 5′ untranslated region (5′-UTR) and a part of open reading frame of *Bmal1* or 5′-UTR of *Rev-erbα* mRNA fused to EGFP (enhanced green fluorescent protein) mRNA. Plasmids encoding fusion mRNAs were transfected into NIH3T3 cells and the effect of polyamines was examined in control and polyamine-reduced cells treated by 1 mM DFMO. We have previously reported that polyamines stimulate Cct2 (T-complex protein 1, β subunit) and COX4 syntheses through the enhancement of ribosome shunting, which involves discontinuous scanning of 5′-UTR by 40S ribosomal subunits [4,5]. For ribosome shunting, take-off and landing sites, which are complementary in sequence to 18S rRNA, are required in the 5′-UTR of mRNA [25]. *Bmal1* mRNA has two hairpin structures: take-off site and landing site in the 5′-UTR of mRNA (Figure 5A). Thus, we made plasmids in which these features required for ribosome shunting to occur were removed. As shown in Figure 5C, polyamine stimulation of BMAL1-EGFP synthesis from native mRNA was observed at a similar extent to BMAL1 synthesis (see Figure 3), whereas polyamine stimulation of BMAL1-EGFP synthesis disappeared and protein synthetic activity increased by removing hairpin 1 and/or hairpin 2. When the nucleotide sequence of 5′-UTR of *Bmal1* mRNA was modified from complementary to non-complementary sequence to the 18S rRNA (NC-18S rRNA), polyamine stimulation of BMAL1-EGFP synthesis disappeared and protein synthetic activity decreased. As a control, synthesis of EGFP (vector) was evaluated, and it was not affected by polyamines. Under these conditions, the level of *Bmal1-EGFP* mRNA did not change in the presence and absence of DFMO (Figure 5D).

**Figure 6:**
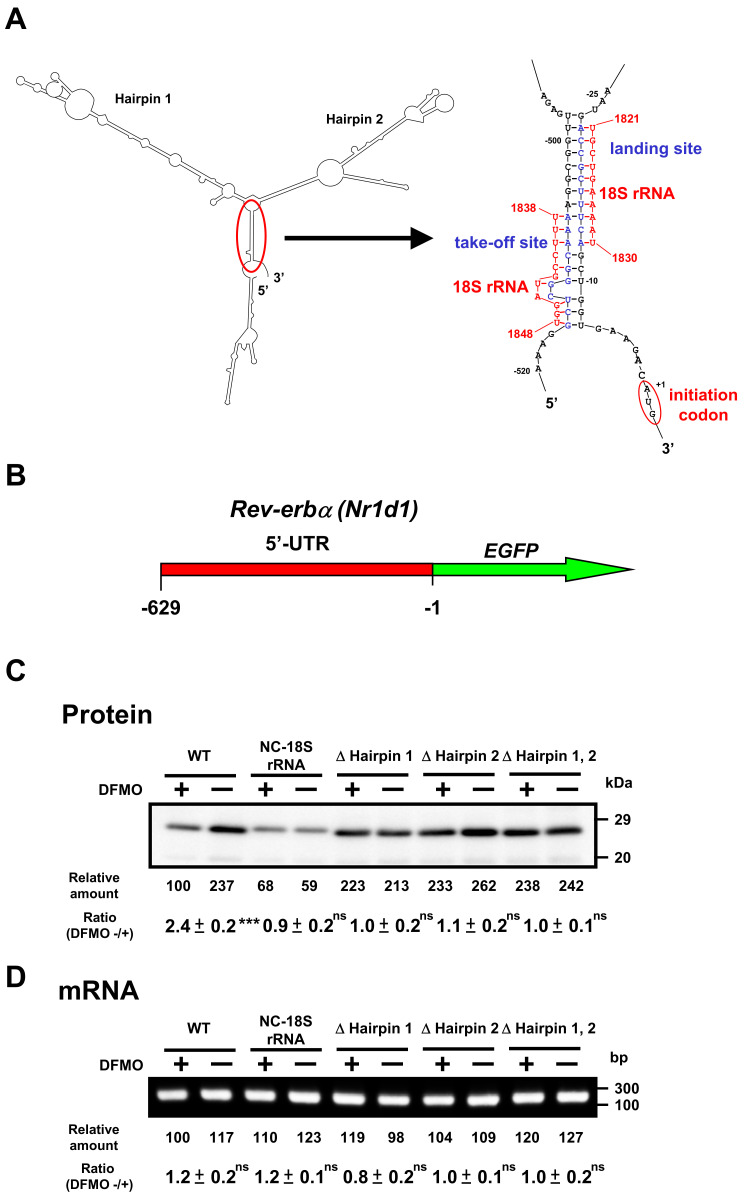
Effect of polyamines on the synthesis of REV-ERBα-EGFP derived from normal and modified *Rev-erbα-EGFP* mRNAs in NIH3T3 cells. Experiments were performed as described in the legend to Figure 5. (**A**) Optimal computer folding of the 5′-untranslated region (UTR) of *Rev-erbα* mRNA was performed by the method of Zuker [26]. Candidates for take-off and landing sites, which are complementary sequence to 18S rRNA are shown in blue, together with the sequence of 18S rRNA in red. (**B**) Structures of *Rev-erbα-EGFP* fusion genes are shown. (**C**,**D**) The levels of protein and mRNA were measured by Western blotting and RT-PCR, respectively. *Rev-erbα-EGFP* fusion gene encoding normal and modified 5′-UTR of *Rev-erbα* mRNA and full length of EGFP protein was transfected into NIH3T3 cells. ns *p* ≥ 0.05; *** *p* < 0.001.

Similarly, we studied the mechanism of polyamine stimulation of REV-ERBα synthesis. Similar to the 5′-UTR of *Bmal1* mRNA, *Rev-erbα* mRNA has two hairpin structures, take-off site and landing site in the 5′-UTR of mRNA (Figure 6A). Since the ribosome shunting is suggested to occur, *Rev-erbα-EGFP* mutants were constructed. As shown in Figure 6C, REV-ERBα-EGFP synthesis from wild-type mRNA was stimulated by polyamines, similar to native REV-ERBα synthesis (see Figure 3), whereas polyamine stimulation of REV-ERBα-EGFP synthesis disappeared and protein synthetic activity increased by removing hairpin structures. When the complementary sequence to 18S rRNA of 5′-UTR of *Rev-erbα* mRNA was converted to non-complementary sequence (NC-18S rRNA), polyamine stimulation of REV-ERBα-EGFP synthesis disappeared and protein synthetic activity decreased. Under these conditions, the level of *Rev-erbα-EGFP* mRNA did not change in the presence and absence of DFMO (Figure 6D). These results suggest that polyamines stimulate the synthesis of BMAL1 and REV-ERBα by the enhancement of ribosome shunting.

## 3. Discussion

**Figure 7:**
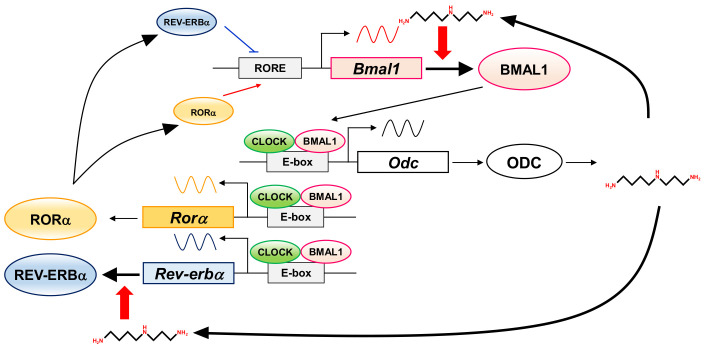
Interrelationship between circadian clock genes and polyamines in the circadian clock. The figure summarizes how circadian clock genes and polyamines are involved.

In this study, to elucidate the role of polyamines in the circadian clock at the molecular level, we looked for clock genes whose synthesis is stimulated by polyamines. The circadian rhythm of intracellular polyamines has been reported in mice, and it was also confirmed in cell culture systems [21]. Here, we show that intracellular polyamine contents are rhythmic (Figure 1). Expression and activity of the polyamine biosynthetic enzymes (ODC, AdoMetDC1, and SRM) become non-rhythmic in various clock mutant mice [17,18]. In particular, ODC, the rate-limiting enzyme for polyamine biosynthesis, is transcriptionally regulated by CLOCK/BMAL1 complex binding to the E-box in the promoter region [20,21]. At the molecular level, circadian rhythms are generated by the interacting transcription/translation feedback loops of the clock genes [12]. CLOCK/BMAL1 also induces nuclear receptors, ROR activators (RORα, RORβ, and RORγ) and REV-ERB repressors (REV-ERBα and REV-ERBβ). These nuclear receptors regulate Bmal1 expression transcriptionally through retinoic acid receptor-related orphan receptor elements (ROREs), thereby constituting an important interlocking feedback loop [14,15]. We show that polyamines enhanced the synthesis of BMAL1 and REV-ERBα at the translational level, i.e., the genes encoding *Bmal1* and *Rev-erbα* are the members of polyamine modulon in eukaryotes. Translational regulation of BMAL1 by polyamines affected a group of genes downstream of *Bmal1*. In fact, the level of *Rorα* was increased at the transcriptional level by polyamines, and the rhythmic phases of *Rev-erbα* and *Per2* were delayed in the absence of polyamines (Figure 2). However, there were some genes (such as CRYs) that were less affected by BMAL1, which was stimulated by polyamines. It is suggested that there are not only circadian clock genes but also other factors in the regulation of the circadian rhythms. Polyamines are at least one of them. Recently, it was reported that Prader–Willi syndrome (PWS)-associated protein necdin regulates BMAL1 stability and circadian clock through chaperone machinery [27]. Accordingly, from our results it can be proposed that the circadian clock is regulated by the interlocked transcriptional/translational feedback loops consisting of clock genes and polyamines (Figure 7). Polyamines are a novel factor in the regulation of the circadian rhythms. Our findings also support the report by Zwighaft et al. that the decline in polyamine levels with age in mice is associated with a longer circadian period [21]. There is no doubt that our health is impaired by either a decrease in polyamines or a decay in circadian clock’s function. 

We studied the mechanism of polyamine stimulation of BMAL1 and REV-ERBα synthesis. The majority of mRNAs in eukaryotic cells are translated via m^7^G cap-dependent and scanning-dependent initiation mechanism. Ribosome shunting is another cap-dependent but non-canonical translation mechanism [25]. Three structural elements are necessary in the 5′-UTR of mRNA for ribosome shunting: a region of stable structure, potential take-off sites, and landing sites. Analysis of predicted secondary structures revealed a similarity to structural elements required for ribosome shunting in the 5′-UTR of *Bmal1* and *Rev-erbα* mRNAs. When these elements were removed from the 5-UTR of these mRNAs, polyamine enhancement of EGFP fusion proteins disappeared (Figure 5 and Figure 6). Thus, the mechanism of polyamine stimulation of BMAL1 and REV-ERBα synthesis is the enhancement of ribosomal shunting, the mechanism similar to that of Cct2 (mammalian cells) and COX4 (yeast) [4,5]. The genes encoding *Bmal1* and *Rev-erbα* are members of polyamine modulon, which encodes proteins whose synthesis is enhanced by polyamines at the level of translation.

Several mechanisms of polyamine stimulation have been uncovered [4,5,6,7,8,9,10]. These publications and this study make it clear that polyamines enhance the synthesis of specific proteins. Polyamines play important roles in physiological phenomena, such as the circadian clock. The further discovery of a polyamine modulon in eukaryotes will clarify the physiological functions of polyamines at the molecular level. In actuality, the physiological functions of polyamines are mainly explained by the functions of 20 members of polyamine modulon in *Escherichia coli* [28].

## 4. Materials and Methods

### 4.1. Cell Culture of Murine Fibroblast NIH3T3 Cells

Mouse fibroblast NIH3T3 cells (3 × 10^5^/10 mL) were cultured in Dulbecco’s modified Eagle’s medium (DMEM) (Wako) supplemented with 100 μg/mL streptomycin, 100 units/mL penicillin G, and 10% heat-inactivated fetal bovine serum (FBS) at 37 °C in an atmosphere of 5% CO_2_ in air. Cells were seeded in 100 mm culture dishes at ~50% confluence and synchronized with 100 nM dexamethasone (Dex) treatment for 2 h [21]. To establish polyamine-reduced NIH3T3 cells, we added 1 mM DFMO (α-difluoromethylornithine, an inhibitor of ornithine decarboxylase), to the medium at the time of seeding.

### 4.2. Preparation of Cell Lysate and Measurement of Polyamines in NIH3T3 Cells

Polyamine contents in NIH3T3 cells were determined according to the method described previously [29]. Control and polyamine-reduced NIH3T3 cells (2 × 10^6^ cells) were treated with 200 μL of 5% (*w*/*v*) trichloroacetic acid (TCA), and centrifuged at 12,000× *g* for 10 min. The supernatant was measured by using a HITACHI HPLC system. The precipitate was dissolved with 100 μL of a buffer containing 25 mM Tris-HCl (pH = 6.8), 1% 2-mercaptoethanol, 5% glycerol, and 1% SDS, and pH of the homogenate was adjusted to pH = 6.8 by 1 M Tris-HCl (pH = 8.0). After standing at room temperature overnight, supernatant was obtained by centrifugation at 17,000× *g* for 15 min and used as a cell lysate. Protein content was determined by the method of Bradford [30].

### 4.3. RNA Isolation and Measurement of the Level of mRNA by Semi-Quantitative Real-Time PCR

Total RNA was extracted from control and DFMO-treated NIH3T3 cells by NucleoSpin RNA (TaKaRa). Complementary DNA was synthesized using a cDNA synthesis kit (ReverTra-Plus-^TM^, TOYOBO), and a semi-quantitative real-time PCR was performed using EmeraldAmpMax PCR Master Mix (TaKaRa) and specific primers (Table S1)—95 °C for 5 min, 25 cycles of (95 °C for 30 s, 55 °C for 30 s, and 72 °C for 30 s), and 72 °C for 5 min according to the accompanying manuals. The PCR product was separated by agarose-gel electrophoresis and stained with ethidium bromide, and band intensity was quantified by a LAS-3000 luminescent image analyzer (Fuji Film). Levels of mRNAs were normalized to *β-actin* mRNA.

### 4.4. Western Blot Analysis

Western blot analysis was performed by the method of Nielsen et al. [31], using horseradish peroxidase-conjugated anti-rabbit IgG (GE Healthcare Bio-Sciences) as secondary antibody and ECL Western blotting reagents (GE Healthcare Bio-Sciences). Antibody against BMAL1 was purchased from Bethyl Laboratories, Inc. Antibodies against CLOCK and REV-ERBα were from Cell Signaling Technology. Antibody against CRY1 was from MEDICAL AND BIOLOGICAL LABORATORIES CO., LTD. Antibodies against PER1, CRY2, and RORα were from Abcam. Antibody against PER2 was from Thermo Fisher Scientific. Antibody against EGFP was from Clontech. The level of protein on the blot was quantified with a LAS-3000 luminescent image analyzer (Fuji Film).

### 4.5. Plasmids

To construct plasmid pBmal1-EGFP(WT), we performed PCR by using primers P19 and P20 (Table S1) and the synthesized cDNA as templates. The amplified *Bmal1* cDNA (a 520-nucleotide 5′-UTR and an 84-nucleotide open reading frame) was digested with EcoRI and SalI, and inserted into the same restriction sites of plasmid pEGFP-N1 (Clontech). Plasmids pBmal1-EGFP(ΔHairpin 1), pBmal1-EGFP(ΔHairpin 2), pBmal1-EGFP(ΔHairpin 1, 2), and pBmal1-EGFP(NC-18S rRNA) were prepared by the site-directed mutagenesis employing overlap extension using PCR [32], and first PCR primer sets were P19 and P22: P16 and P21 for pBmal1-EGFP(ΔHairpin 1), P19 and P24: P20 and P23 for pBmal1-EGFP(ΔHairpin 2), P19 and P26: P20 and P25 for pBmal1-EGFP(ΔHairpin 1, 2), P19 and P28: P20 and P27 for pBmal1-EGFP(NC-take off site), and P19 and P30: P20 and P29 for pBmal1-EGFP(NC-18S rRNA). pBmal1-EGFP(WT) was used as a template. pBmal1-EGFP(ΔHairpin 1) was used as a template to construct pBmal1-EGFP(ΔHairpin 1, 2). pBmal1-EGFP(NC-take off site) was used as a template to construct pBmal1-EGFP(NC-18S rRNA). Primers P19 and P20 were used for second PCR. The second PCR product was digested with EcoRI and SalI, and inserted into the same restriction sites of plasmid pEGFP-N1. Plasmids pRev-erbα-EGFP(WT) (a 629 nucleotide 5′-UTR) and modified plasmids were similarly constructed. The nucleotide sequence of the plasmids was confirmed by the 3130 Genetic Analyzer (Applied Biosystems) using P41 as a primer.

### 4.6. Transient Transfection of Fusion Plasmids into NIH3T3 Cells and Measurement of Their Protein Levels

NIH3T3 cells (3 × 10^5^/10 mL) were cultured in DMEM supplemented with 50 units/mL streptomycin, 100 units/mL penicillin G, and 10% FBS at 37 °C in an atmosphere of 5% CO_2_ in air for 36 h. Then, cells were cultured in the presence and absence of 1 mM DFMO for 12 h. After changing the medium with a fresh one without FBS, we transfected cells with 4 μg of various fusion plasmids by Lipofectamine 2000 Reagents (Thermo Fisher Scientific) according to the manufacturer’s instructions and cultured for 4–6 h. After changing the medium with a fresh one containing FBS, we cultured cells in the presence and absence of 1 mM DFMO for a further 24 h. NIH3T3 cells attached to the culture dish were washed twice with 5 mL of phosphate-buffered saline (PBS) and incubated with 0.4 mL of 0.25% trypsin–0.02% EDTA-4Na solution at 37 °C for 3 min, and then 5 mL of DMEM containing 10% FBS was added to the culture dish. Dispersed cells were collected by centrifugation at 300× *g* for 5 min, washed twice with PBS, and used for Western blotting as described above.

### 4.7. Statistics

Values are indicated as mean ± SE (Standard Error) of triplicate determinations. Data of control and DFMO-treated groups were analyzed by Student’s *t* test, and a statistical difference was shown by probability values.

## Data Availability

All data are contained within the manuscript.

## Supplementary Materials

The following are available online at https://www.mdpi.com/1422-0067/22/3/1307/s1: Table S1: Primers used in this study.

## Abbreviations

AdoMetDC1	*S*-adenosylmethionine decarboxylase 1
Dex	dexamethasone
DFMO	α-difluoromethylornithine
EDTA	ethylenediaminetetraacetic acid
ns	not significant
ODC	ornithine decarboxylase
PUT	putrescine
SE	standard error
SPD	spermidine
SPM	spermine
SRM	spermidine synthase
5′-UTR	5′-untranslated region
