# Supplementary material for: Translational Regulation of Clock Genes BMAL1 and REV-ERBα by Polyamines
Source: Int J Mol Sci. 2021 Jan 28;22(3):1307. doi: 10.3390/ijms22031307 (PMC7865260; doi:10.3390/ijms22031307)
Supplement: Supplementary file 1 [file ijms-22-01307-s001.zip › IJMS Supplementary material Table S1-210112.pdf]

Table S1 Primers used in this study.

No.	Primer used	Nucleotide sequence
P1	5'-β-actin	5'-CAGGTCATCACTATTGGCAACGAGCGGTTC-3'
P2	3'-β-actin	5'-GGAGCCAGAGCAGTAATCTCCTTCTGCATC-3'
P3	5'-mBmal1	5'-TGTCCAGGAAGTTAGATAAACTCACCGTGC-3'
P4	3'-mBmal1	5'-AGTCAAACAAGCTCTGGCCAATAAGGTCAT-3'
P5	5'-mClock	5'-TACTCTCTACTCATCTGCTGGAAAGTGACT-3'
P6	3'-mClock	5'-CTTCATAAGAAGGCCTATGTGTGCGTTGTA-3'
P7	5'-mPer1	5'-GACACTGATGCAAACAGCAATGGCTCAAGT-3'
P8	3'-mPer1	5'-CTGGTAATATTCCTGGTTAGCCTGAACCTG-3'
P9	5'-mPer2	5'-CGGATGCTCGTGGAATCTTCCAACACTCAC-3'
P10	3'-mPer2	5'-CCTGCTCCATGCTGTAGGAAGGCACATCCA-3'
P11	5'-mCry1	5'-TCAGTGTCTTGAGGATCTTGATGCCAATCT-3'
P12	3'-mCry1	5'-GGCTGTCCGCCATTGAGTTCTATGATCTTG-3'
P13	5'-mCry2	5'-AATTCTTCTACACAGCGGCCACCAACAACC-3'
P14	3'-mCry2	5'-CATTCACACTGAAATCGGCATCCAGGAGCA-3'
P15	5'-mRORα	5'- TACTCCTGTCCTCGTCAGAAGAACTGTTTG-3'
P16	3'-mRORα	5'- CAGTTCCGTCAGCCCATTGGCTGAGATGTT-3'
P17	5'-mReverbα	5'-GTGGTGTTATCACCTACATTGGCTCTAGTG-3'
P18	3'-mReverbα	5'-TGTCTTCCATGGCCACTTGTAGACTTCCTG-3'
P19	5'-Bmal1(EcoRI)	5'-ATTAGAATTCGGCCGGGCCTGGGCCGGCGG-3'
P20	3'-Bmal1(Sal1)	5'-ACCACTGGTCGACAGGGAACCGGAGAGTAG-3'
P21	5'-Bmal1(ΔHairpin 1)	5'-GGCAGAAAGTAGCGAAGCTTAAGAACTG-3'
P22	3'-Bmal1(ΔHairpin 1)	5'-CTTAAGCTTCGCTACTTTCTGCCTTCCCTA-3'
P23	5'-Bmal1(ΔHairpin 2)	5'-ACTGGATCGAAGCACCTTCCTTC-3'
P24	3'-Bmal1(ΔHairpin 2)	5'-GAAGGAAGGTGCTTCGATCCAGT-3'
P25	5'-Bmal1(ΔHairpin 1,2)	5'-GCAGAAAGTAGCGAAGCACCTTCCTTCCAA-3'
P26	3'-Bmal1(ΔHairpin 1,2)	5'-TTGGAAGGAAGGTGCTTCGCTACTTTCTGC-3'
P27	5'-Bmal1(NC take off)	5'-GTGCGACATTATGCCTTCGCAGAAAGTAGG-3'
P28	3'-Bmal1(NC take off)	5'-CCTACTTTCTGCGAAGGCATAATGTCGCAC-3'
P29	5'-Bmal1(NC landing)	5'-CTTGCAAGCACGAAGGAACCAATGGCGGAC-3'
P30	3'-Bmal1(NC landing)	5'-GTCCGCCATTGGTTCCTTCGTGCTTGCAGG-3'
P31	5'-Reverb(EcoRI)	5'-ATATGAATTCACTGGGGCACGAGGCGCTCCCTGGAATCACATGGT-3'
P32	3'-Reverb(BamHI)	

		5’-CGCAGGATCCGTCTTCACCAGCTGAAAGCGGTCATTCAAACTGGA-3’
P33	5’-Reverb(ΔHairpin 1)	5’-CGGTTGAGATTGGCAGCTGCTGCAGGC-3’
P34	3’-Reverb(ΔHairpin 1)	5’-GCCTGCAGCAGCTGCCAATCTCAACCG-3’
P35	5’-Reverb(ΔHairpin 2)	5’-CTGCTGCAGGCGTTTGAATGACCGCTTTC-3’
P36	3’-Reverb(ΔHairpin 2)	5’-GAAAGCGGTCATTCAAACGCCTGCAGCAG-3’
P37	5’-Reverb(NC take off)	5’-CCAGGGAAAGTGGATGCCTTTAGGCGGTTG-3’
P38	3’-Reverb(NC take off)	5’-CAACCGCCTAAAGGCATCCACTTTCCCTGG-3’
P39	5’-Reverb(NC landing)	5’-GTTTGAATGTGCTGAAAATGCTGGTGAAG-3’
P40	3’-Reverb(NC landing)	5’-CTTCACCAGCATTTTCAGCACATTCAAAC-3’
P41	5’-pEGFP (seq)	5’-GGACTTTCCATAATGTCGTAACAACTCCGC-3’
