# Supplementary material for: Translational Regulation of Clock Genes BMAL1 and REV-ERBα by Polyamines
Source: Int J Mol Sci. 2021 Jan 28;22(3):1307. doi: 10.3390/ijms22031307 (PMC7865260; doi:10.3390/ijms22031307)
Supplement: Supplementary file 1 [file ijms-22-01307-s001.zip › Original Files of Western blot-210112.pptx]

## Slide 1
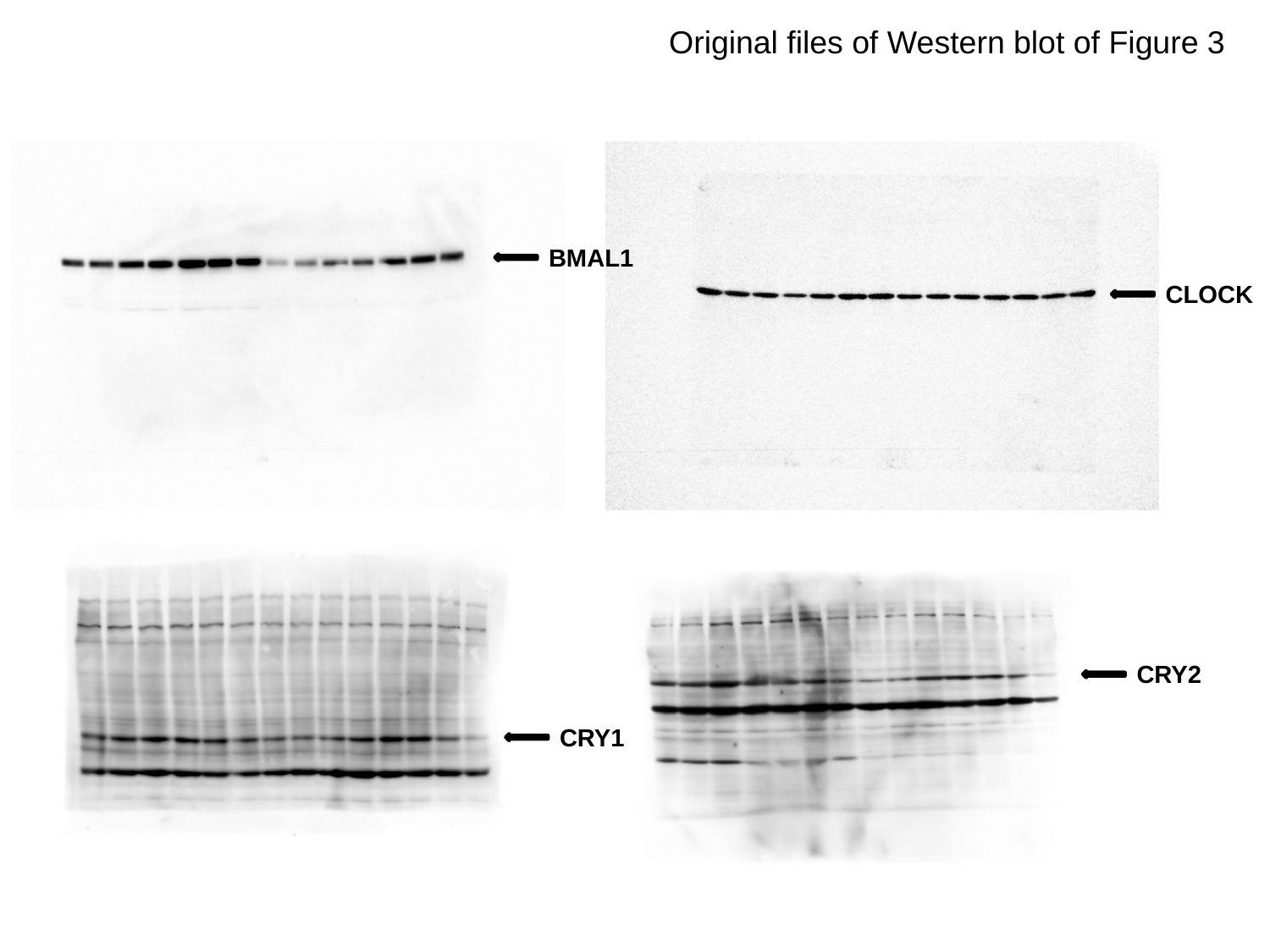

Original files of Western blot of Figure 3
BMAL1
CLOCK
CRY2
CRY1

## Slide 2
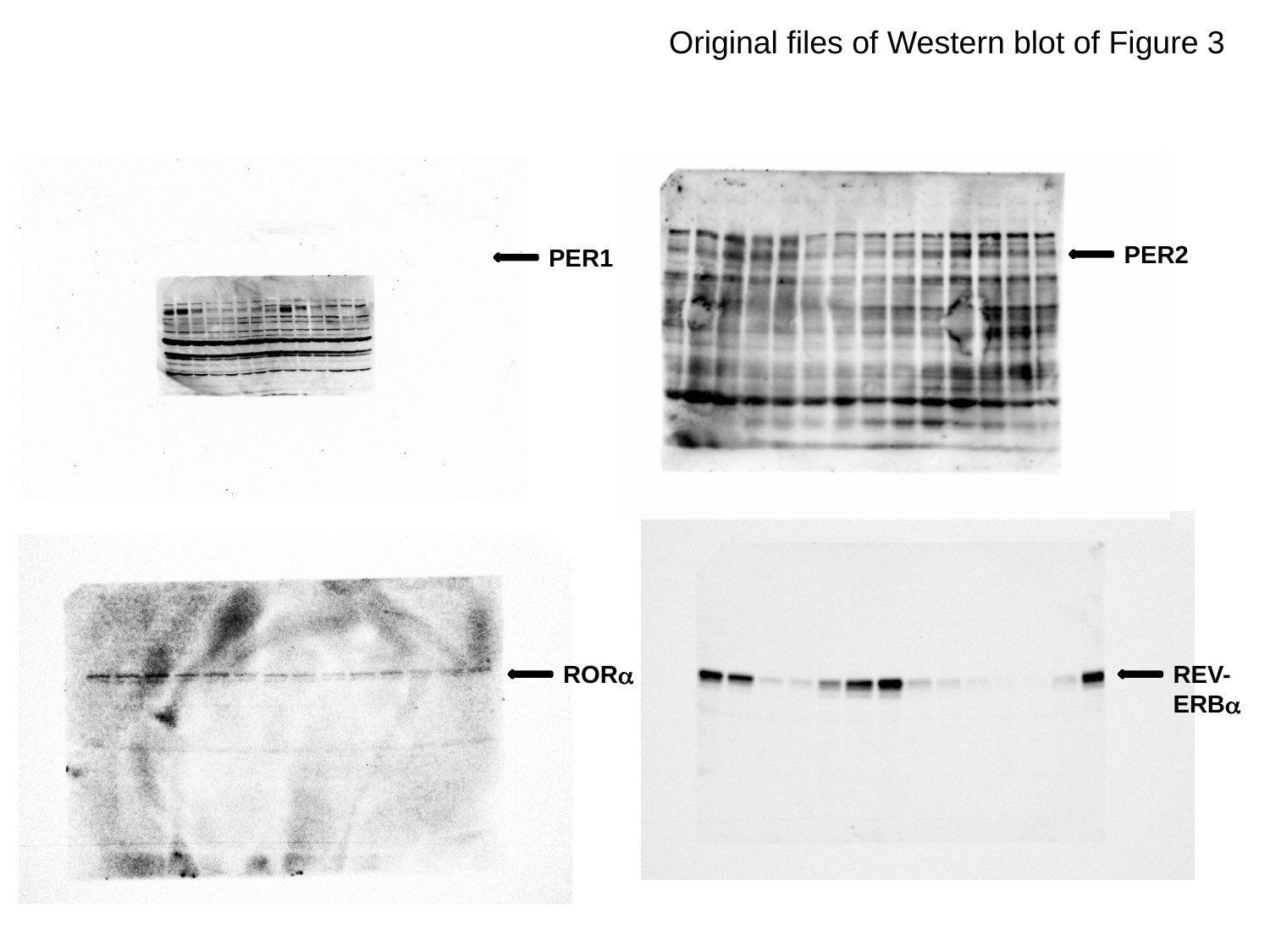

Original files of Western blot of Figure 3
PER2
PER1
RORa
REV-ERBa

## Slide 3
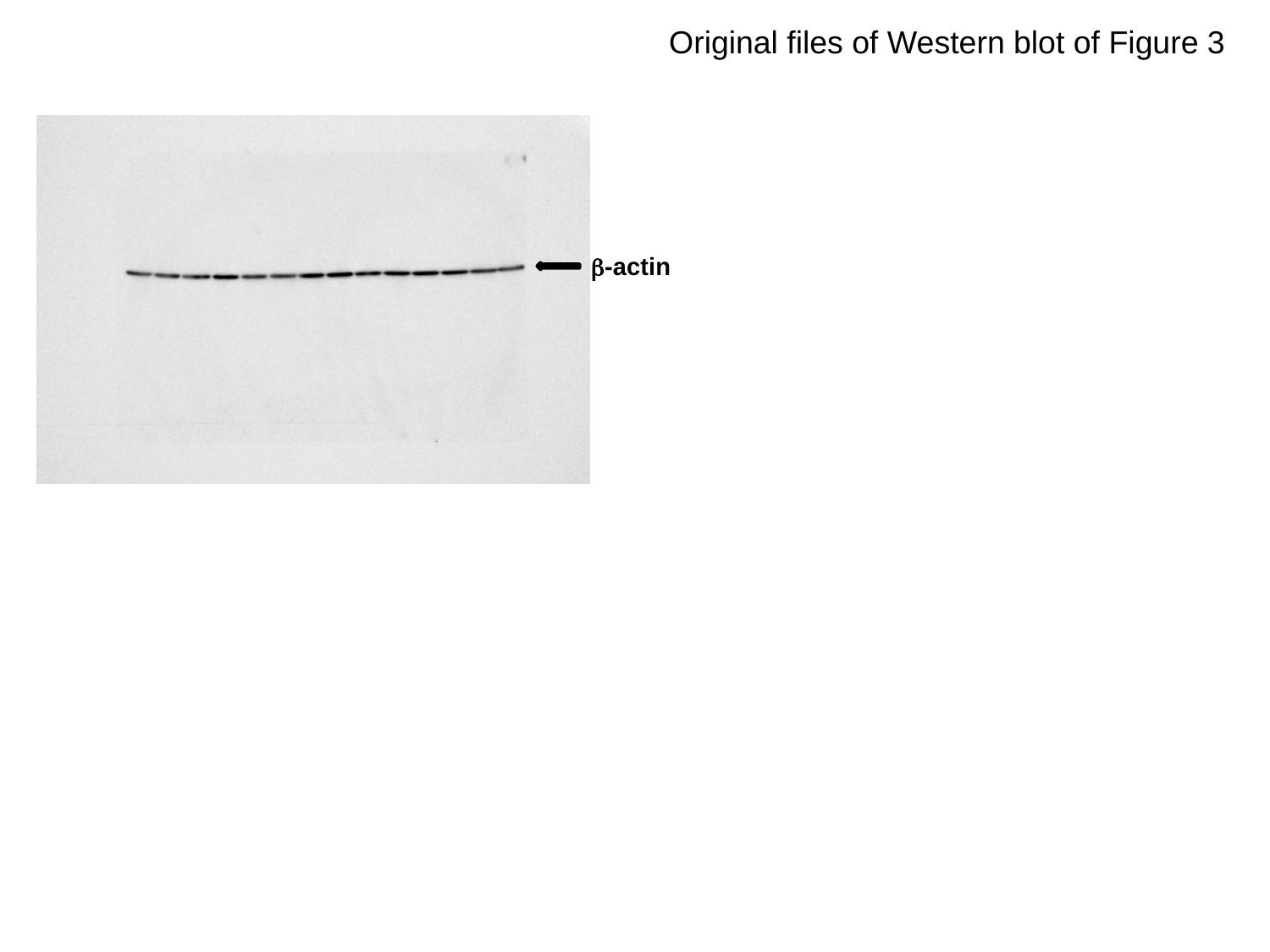

Original files of Western blot of Figure 3
b-actin

## Slide 4
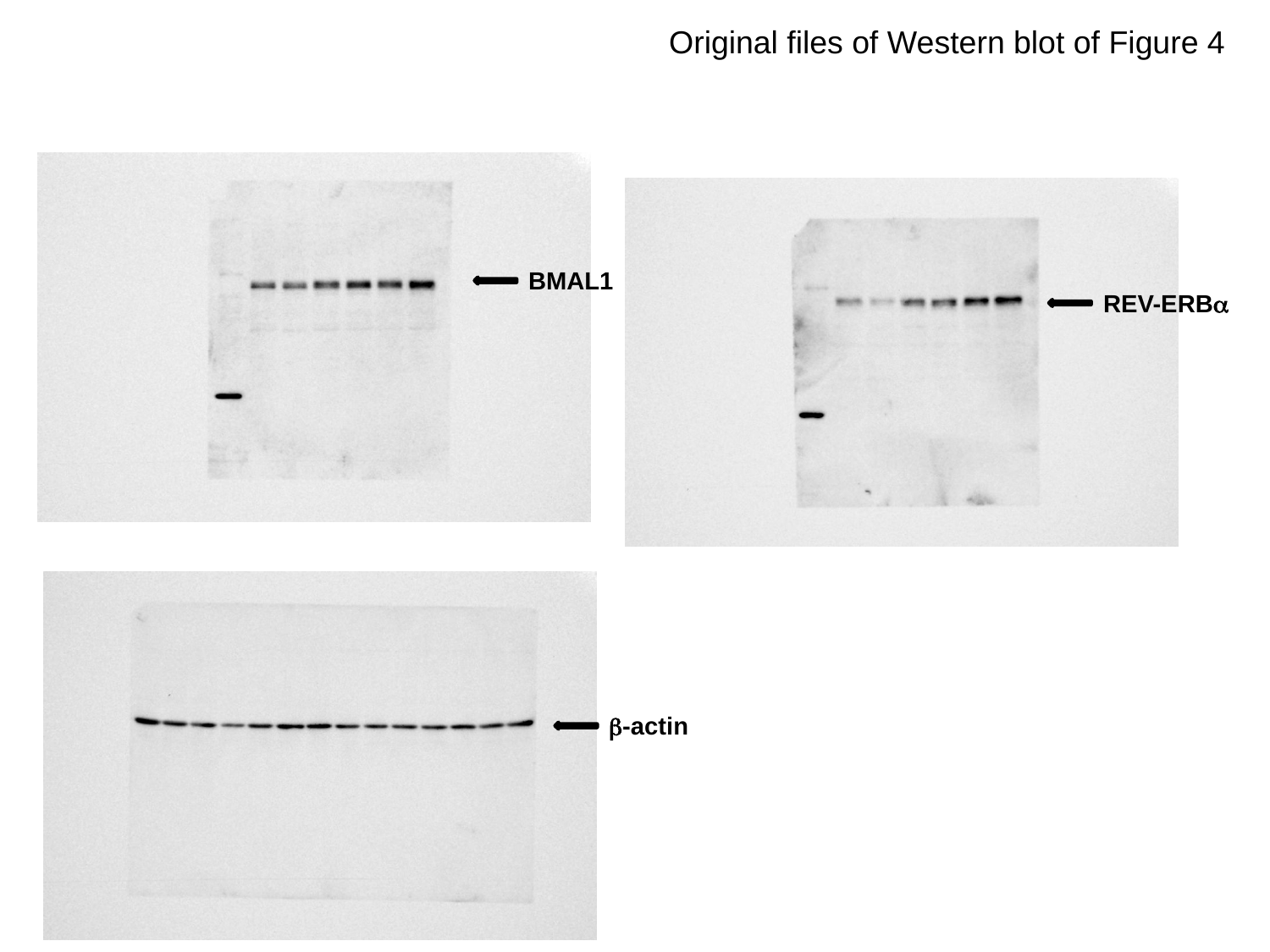

Original files of Western blot of Figure 4
BMAL1
REV-ERBa
b-actin

## Slide 5
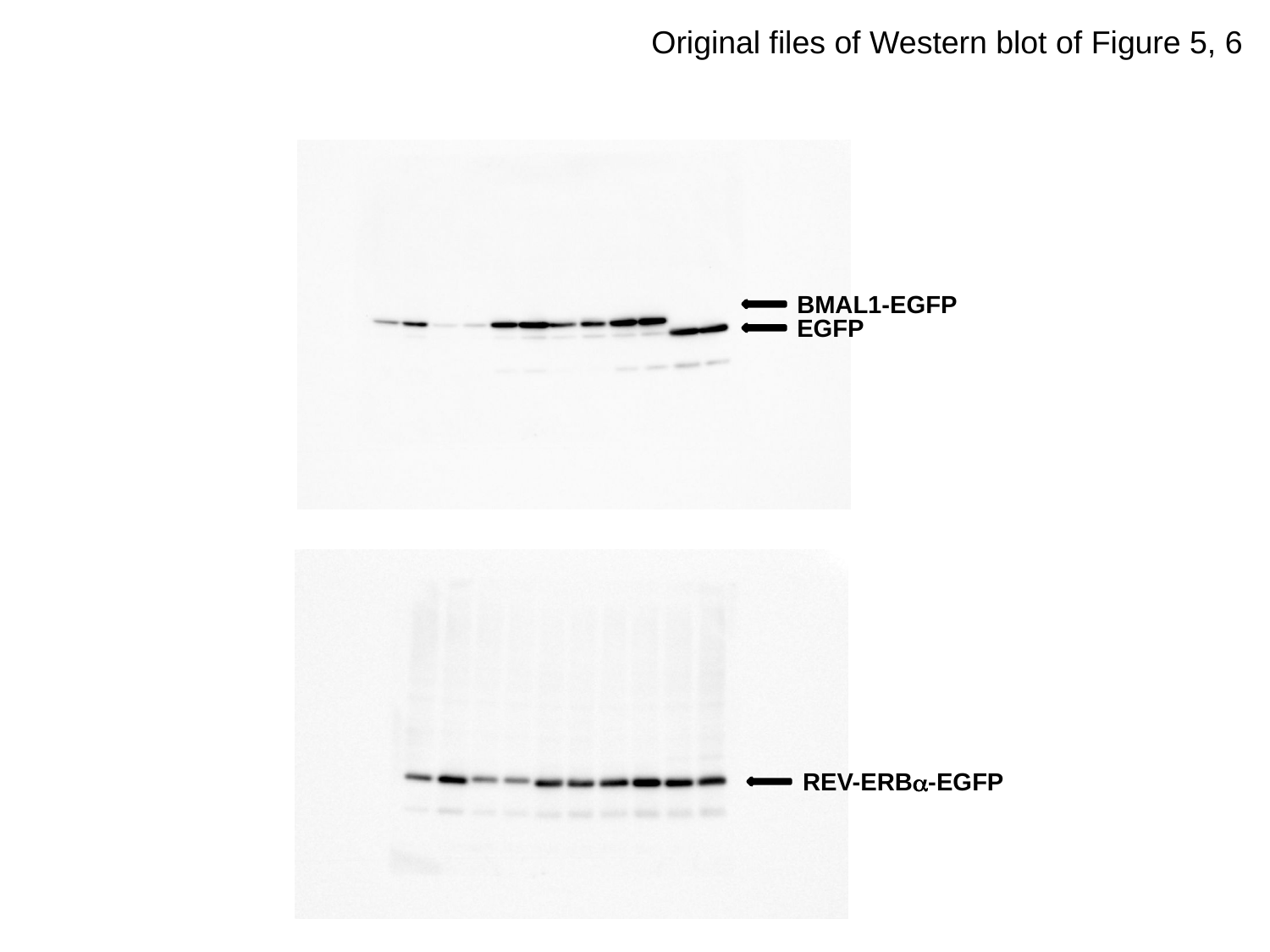

Original files of Western blot of Figure 5, 6
BMAL1-EGFP
EGFP
REV-ERBa-EGFP
